# Morphometry and Contents of the Suprascapular Notch with Potential Clinical Implications: Α Cadaveric Study

**DOI:** 10.1055/s-0041-1731749

**Published:** 2021-07-27

**Authors:** George Tsikouris, Ioannis Antonopoulos, Dionysia Vasdeki, Dimosthenis Chrysikos, Athanasios Koukakis, George Tsakotos, Panagiotis Georgakopoulos, Theodore Troupis

**Affiliations:** 1 Department of Anatomy, School of Medicine, National and Kapodistrian University of Athens, Athens, Greece

**Keywords:** suprascapular notch, suprascapular nerve, neuropathy, morphometry, shoulder surgery, fresh frozen, cadavers

## Abstract

**Background** The suprascapular notch (SN) represents the point along the route of the suprascapular nerve (SSN) with the greatest potential risk for injury and compression. Thus, factors reducing the area of the notch have been postulated for suprascapular neuropathy development.

**Methods** Thirty-one fresh-frozen shoulders were dissected. The contents of the SN were described according to four types as classified by Polguj et al and the middle-transverse diameter of the notch was measured. Also, the presence of an ossified superior transverse scapular ligament (STSL) was identified.

**Results** The ligament was partially ossified in 8 specimens (25.8%), fully ossified in 6 (19.35%), and not ossified in the remaining 17 (54.85%). The mean middle-transverse diameter of the SN was 9.06 mm (standard deviation [SD] = 3.45). The corresponding for type-I notches was 8.64 mm (SD = 3.34), 8.86 mm (SD = 3.12) was for type-II, and 14.5 mm (SD = 1.02) was for type III. Middle-transverse diameter was shorter when an ossified ligament was present (mean = 5.10 mm, SD = 0.88 mm), comparing with a partially ossified ligament (mean =7.67 mm, SD = 2.24 mm) and a nonossified one (mean = 11.12 mm, SD = 2.92 mm). No statistically significant evidence was found that the middle-transverse diameter depends on the number of the elements, passing below the STSL.

**Conclusion** Our results suggest that SSN compression could be more likely to occur when both suprascapular vessels pass through the notch. Compression of the nerve may also occur when an ossified transverse scapular ligament is present, resulting to significant reduction of the notch's area.

## Introduction

The suprascapular nerve (SSN) is a mixed peripheral nerve that originates from the superior trunk of the brachial plexus and innervates the supraspinatus and infraspinatus muscles, its sensory components supply the acromioclavicular and glenohumeral joints, and there have been also described lateral and medial subacromial branches coming from it. 1 2 3 4 It typically stems from the fifth and sixth cervical nerves, although a contribution from the fourth cervical nerve has also been identified. SSN runs through the posterior triangle of the neck, underneath the trapezius, and parallel to the omohyoid muscles. It enters the supraspinous fossa through the suprascapular notch (SN), running under the superior transverse scapular ligament (STSL), then passes deep to the supraspinatus muscle and after passing through the spinoglenoid notch, it reaches the infraspinous fossa.

The SN is a bony depression in the lateral part of the superior border of the scapula, situated medially to the coracoid process. The SN is normally bridged by the STSL, creating an osteofibrous tunnel. STSL may be ossified, converting so the SN into an osseous foramen, causing stenosis of the notch and possible compression of the nerve. Ossification of STSL ranges from 0.3 to 13.6%, and its frequency varies according to the region. 1 5 6

SSN constantly runs through the SN, most commonly accompanied by the suprascapular vein, while the suprascapular artery runs over the STSL. However, the courses of the associated suprascapular vessels are subject to significant variation. 7 8 9

The suprascapular vein starts in the infraspinatus fossa, after passing through the spinoglenoid notch enters the supraspinous fossa, and following ascends to the SN. It most commonly crosses the SN running under the STSL along with the SSN and then travels with the suprascapular artery and finally empties into the external jugular vein. 10 It is, though, common the SSV's course over the STSL.

The suprascapular artery derives from first part of the subclavian artery, most commonly as a branch of the thyrocervical trunk. Less frequently, it may originate from the internal thoracic artery (1–1.5%), the costocervical trunk (1%), or the dorsal scapular artery. 11 It runs laterally and anteriorly to the anterior scalene muscle, crosses the proximal brachial plexus and the subclavian artery, and then travels parallel with the clavicle. After passing through the inferior belly of the omohyoid muscle, it reaches the superior margin of the scapula and enters the supraspinous fossa passing above the STSL to supply the supraspinatus muscle. 12 13 This course of the suprascapular artery can help the surgeon identify it and then the SSN that will usually be in an inferior surgical plane. However, there have been described cases in which the artery was passing beneath the STSL, along with the SSN. In these cases, the variant course of the artery was accompanied by a variation of its origin, arriving from either the first part of the axillary artery or the third part of the subclavian artery. 11

The SN represents the point along the route of the SSN with the greatest potential for its injury and compression, therefore it is a region of paramount importance for the development of suprascapular neuropathy. 5 14

Suprascapular neuropathy is a symptomatic syndrome, affecting mainly patients under the age of 35 years, presenting as dull with chronic pain of the shoulder associated with weakness of the arm. 1 8 14 It has been estimated that of all shoulder pains in the whole population, 1 to 2% are due to this syndrome, while in international-level high-performance volleyball players, its occurrence can be as high as 33%. 1 2 5

Many factors have been associated with the etiopathogenesis of suprascapular neuropathy. Anatomical variations of the structures forming this osteofibrous tunnel, the shape and size of the notch or direct compression of the nerve by a mass in this region (e.g., ganglion/labral cyst and tumor) may be the cause of SN neuropathy. Moreover, repetitive irritation in overhead activities, continuous traction secondary to a rotator cuff tear, or inflammation of the nerve can all result in neuropathy through the “sling effect” which means pressure of a sharply shaped SN's border on the SSN. 1 6 8 15 16 Ossification of the STSL has also been implicated with the development of SSN entrapment syndrome. 8 15

In the present study, we describe our findings after dissection of 31 fresh-frozen cadaveric shoulders. Anatomical variations, middle-transverse diameter of the SN, and ossification of the STSL were recorded. Correlations of the results of our measurements with aim to reveal potential causes of SN stenosis and SSN entrapment was attempted.

## Materials and Methods

Thirty-one fresh-frozen cadaveric shoulders were dissected at the Dissections' Hall of Department of Anatomy of Medical School in the University of Athens. Each cadaveric material used in this study derived from body donation, with informed consent signed by the donor. 17 In all donors, a horizontal incision along the clavicle was performed initially. Following, the skin was separated from the trapezius, deltoid, and pectoralis major muscles. Trapezius and deltoid muscles were disinserted, so that the supraspinatus, infraspinatus, and subscapularis muscles to get exposed. Retraction of the supraspinatus muscle from the supraspinatus fossa allowed visualization of the SN. The content of the SN was described and the arrangement of the corresponding nerve, artery, and vein in relation to the STSL was noted according to classification by Polguj et al, since it is a simple, reproducible system based on geometrical measurements. 9

Furthermore, the presence of an ossified STSL was anatomically evaluated via direct visualization after cleaning the STSL from the surrounding soft and fat tissue and for the hardness offered during its cut. The extent of the STSL's ossification was categorized as either “partial” or “full” based on the length of the STSL being ossified. A “fully ossified” STSL was ossified all across its length, forming an osseous foramen. Α “partially ossified” STSL was only at a portion of it ossified. No histological stain was used.

**Fig. 1:**
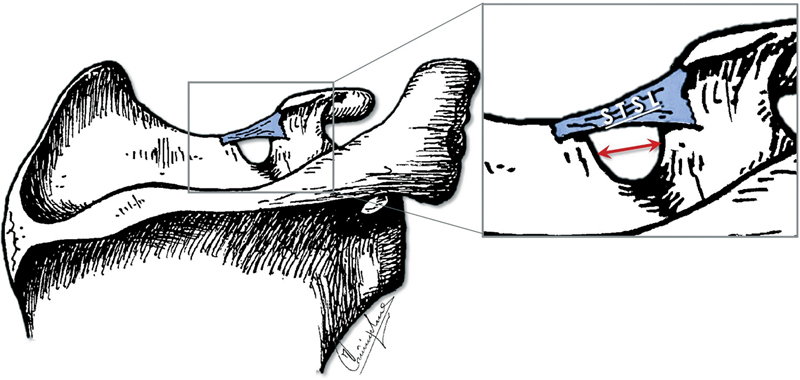
Illustration of the scapula's upper part, showing the distance measured as the SN's middle-transverse diameter (red double arrow). SN, suprascapular notch; STSL, superior transverse scapular ligament.

In addition, the width (middle-transverse diameter) of the SN was measured as the maximal distance between its lateral and medial borders ( Fig. 1 ). The measurements were made using a Würth digital Vernier caliper (accuracy of 0.01 mm) and recorded in the form of tables. For statistical analysis, STATA MP13 was used. Statistically significant difference was considered if *p* < 0.05.

## Results

**Fig. 2:**
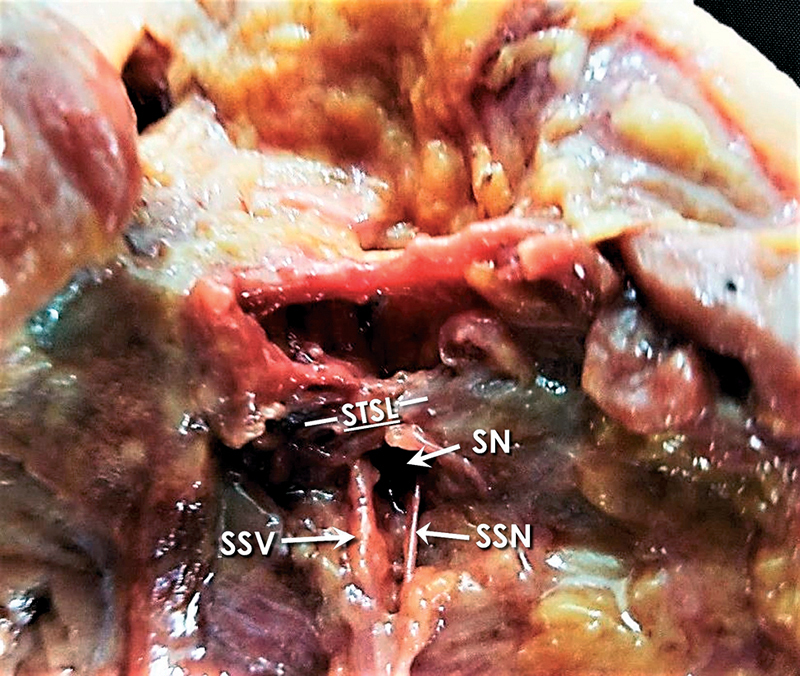
Type-I content of the SN according to the classification used (normal anatomy). SN, suprascapular notch; SSN, suprascapular nerve; SSV, suprascapular vein; STSL, superior transverse scapular ligament.

**Fig. 3:**
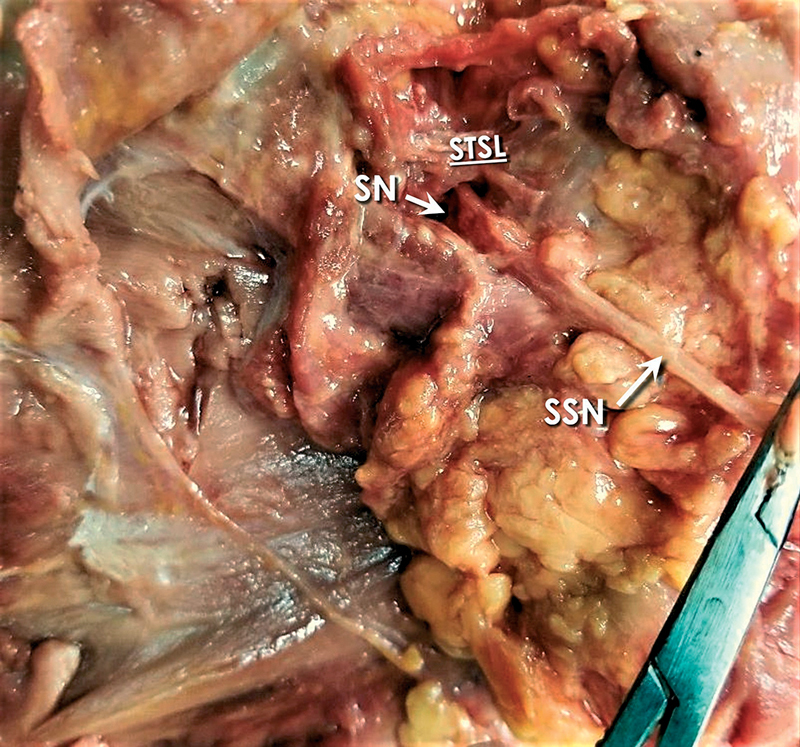
Type-II content of the SN according to the classification used (only the nerve through the notch). SN, suprascapular notch; SSN, suprascapular nerve; SSV, suprascapular vein; STSL, superior transverse scapular ligament.

**Fig. 4:**
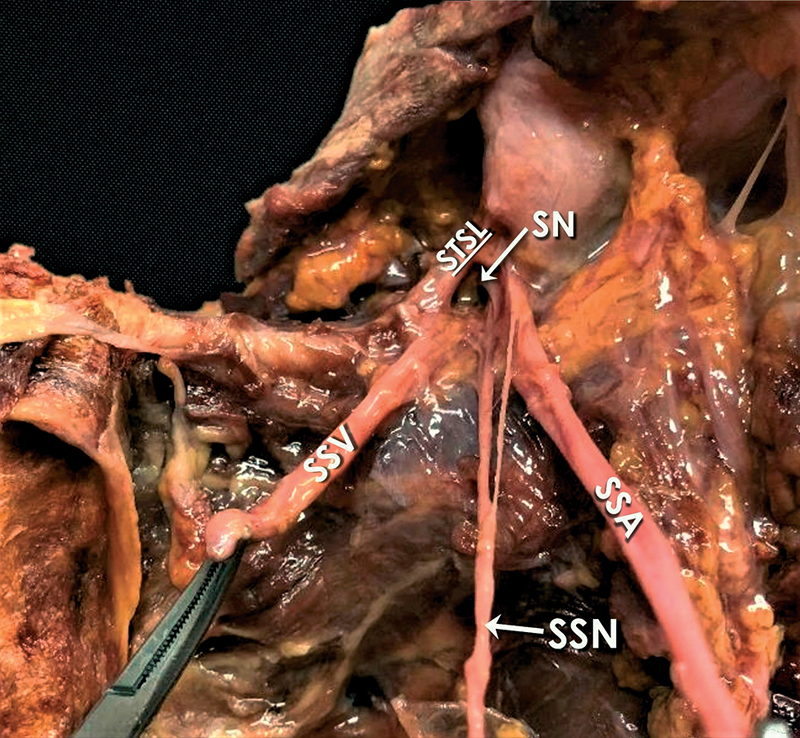
Type-III content of the SN according to the classification used (nerve and both vessels through the notch). SSA, suprascapular artery; SN, suprascapular notch; SSN, suprascapular nerve; SSV, suprascapular vein; STSL, superior transverse scapular ligament.

**Fig. 5:**
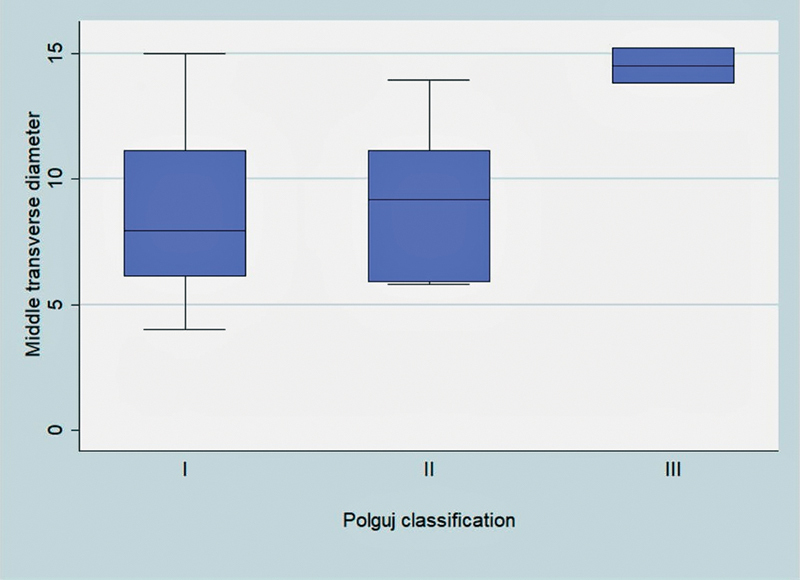
Variance of SN middle-transverse diameter depending on SN content type according to Polguj's classification.

In all specimens, the SSN travelled below the STSL. Polguj et al in 2013 suggested a classification system regarding the arrangement of the SSN, artery, and vein at the SN. As per this classification system, there are four types describing the topography of these elements at the SN. In type I, the artery runs above the STSL, while the nerve and the vein pass beneath the ligament ( Fig. 2 ). In type II, only the nerve passes under the ligament, while both vessels cross over it ( Fig. 3 ). In type III, suprascapular artery, vein, and nerve pass under the ligament ( Fig. 4 ); and in type IV, all other variants of these structures are involved. 9 In our study, 22 of 31(70.97%) shoulders were classified as type I, 7 of 31 (22.58%) shoulders as type II, 2 of 31 (6.45%) as type III, and none (0%) as type IV. The mean middle-transverse diameter of the SN was 9.06 mm (standard deviation [SD] = 3.45). The corresponding for type-I notches was 8.64 mm (SD = 3.34), 8.86 mm (SD = 3.12) was for type II, and 14.5 mm (SD = 1.02) was for type III ( Table 1 ). One-way analysis of variance (ANOVA) was performed to evaluate if the mean middle-transverse diameters were different among the three groups based on the classification by Polguj et al ( Fig. 5 ). Mean middle-transverse diameter wasn't statistically different among the groups.

**Table 1:** Middle-transverse diameters categorized by SN's content type (classification by Polguj et al)

Scapulae	*n* (%)	Mean (mm)	SD (mm)	Min (mm)	Max (mm)
Type I	22 (70.97)	8.64	3.36	4.02	14.97
Type II	7 (22.58)	8.86	3.12	5.82	13.94
Type III	2 (6.45)	14.05	1.02	13.78	15.22
Total	31 (100)	9.06	3.45	4.02	15.22

Abbreviations: Max, maximum; Min, minimum; SN, suprascapular notch; SD, standard deviation.

The STSL was partially ossified in 8 specimens (25.8%), fully ossified in 6 shoulders (19.35%), and not ossified in the remaining 17 shoulders (54.85%). Middle-transverse diameter was shorter when an ossified STSL was present (mean = 5.10 mm, SD = 0.88 mm), comparing with a partially ossified STSL (mean =7.67 mm, SD = 2.24 mm) and a nonossified STSL (mean = 11.12 mm, SD = 2.92 mm). We have statistically significant evidence at *p* = 0.0001 to show that there is a difference in mean middle-transverse diameters in the scapulae with a fully ossified STSL compared with partially ossified STSL and nonossified STLS. Tukey's pot hoc analysis revealed that the reduction from nonossified STLS to fully ossified STSL (−5.78, 95% confidence interval [CI]: [−8.67, −2.89]) and also the reduction between nonossified STLS and partially ossified STLS (−3.21, 95% CI: [−5.82, −0.6]) were statistically significant ( *p* < 0.05), but no statistically significant difference found between fully ossified STLS and partially ossified STLS ( Table 2 ).

**Table 2:** Results of pairwise comparison of notch transverse diameters regarding STSL ossification status

STSL type	Contrast	SE	Turkey	*p* -Value	95% CI	
Full ossification vs. nonossification	−5.78	1.16	−4.96	0.0001	−8.67	−2.89
Partially ossification vs. nonossification	−3.21	1.05	−3.05	0.014	−5.82	−0.6
Partial vs. full ossification	2.57	1.31	1.95	0.143	−0.7	5.83

Abbreviations: CI, confidence interval; SE, standard error; STSL, superior transverse scapular ligament.

## Discussion

The SN is a very heterogeneous anatomical region, characterized by many anatomical variations, which may predispose to suprascapular neuropathy. Traction injuries, direct trauma, compression by ganglion cysts or tumors, variations in the morphology of neighboring soft tissues, or even a hypertrophied subscapular muscle may contribute to its development. 1 8 14 15 Additionally, suprascapular neuropathy development has been associated with autoinflammatory diseases, specifically rheumatoid arthritis and systematic lupus erythematosus. 18 19 SSN neuropathy has been described as a symptomatic syndrome related with weakness of the arm, difficulty in external rotation, and abduction, followed by atrophy of the infra- and supraspinatus muscles and a deep, dull, and chronic pain in the posterior and lateral aspects of the arm and shoulder, with occasional radiation down to the arm or up into the neck. 1 8 14 It mainly affects males and it occurs in patients younger than 35 years of age. 7 14

Embryologically, all main vessels are derived from a primary plexus of small vessels. During this phase, some vessels enlarge and develop definite channels, while other vessels may regress. Therefore, it is during this phase of the vessels that variations of either the origin or the course of the vessels may occur. 13 20

The route of the SSN through the SN is constant, as it always runs beneath STSL. However, the locations of the corresponding artery and vein vary. 7 There have been two classification systems describing the arrangement of the suprascapular triad at the area of notch by Yang et al in 2012 and Polguj et al in 2013. 4 6 Yang et al distinguished three types based on an examination of 103 shoulders. In type I (60 shoulders; 59.4%), all suprascapular vessels pass over the STSL; in type II (30 shoulders; 29.7%), the vessels cross over and under the STSL simultaneously; in type III (11 shoulders; 10.9%), all vessels run under the STSL. 4 Furthermore, type II has been divided into four subtypes, regarding the location of the suprascapular vein related to the STSL and the presence of additional veins. 4 Polguj et al described four types of suprascapular triad topography. In type I (61.3%), the artery runs above the STSL, while the nerve and the vein pass beneath the ligament. In type II (17%), only the nerve passes under the ligament, while both vessels cross over it. In type III (12.3%), suprascapular artery, vein, and nerve lie under the ligament; and in type IV (9.4%), all other variants of these structures are involved. 9

Our findings were in accordance with the classification of Polguj et al, that is, type I was the commonest type, but our percentages were slightly higher than the ones proposed by Polguj et al. The smaller sample size in the present study and the variance in location of the suprascapular triad depending on the population studied may be the reasons for this difference in percentages.

Complete ossification of the STSL ranges from 0.3 to 13.6%, and the frequency varies according to the region. 6 In our study 6 out of 31 shoulders presented a completely ossified STSL, stating 19.3% of the shoulders were studied. This is a quite high percentage, comparing with other similar studies by Sinkeet et al (3%), Rengachary et al (4%), Agrawal et al (7.14%), Polguj et al (0–7%), Natsis et al (0%), Adewale et al (8%), and/or Bayramoğlu et al (12.5%). 1 2 10 14 16 21 22 23 Furthermore, in our study partial ossification of the STSL was observed in 8 out of 31 scapulae (25.8%) which is higher to the observations made by Agrawal et al (5.76%), Long et al (6%), Dunkelgrun et al (12%), and Ticker et al (18%) 1 8 24 25 but quite similar with the findings by Polguj et al (23.3%). In our study, SN with an ossified STSL presented with a shorter middle-transverse diameter (5.16 ± 0.98 mm) comparing with shoulders with a nonossified STSL (11.12 ± 2.93 mm). A short middle-transverse diameter indicates a narrow notch which may predispose patient to suprascapular neuropathy. 14 It is worth mentioning that ossification of the STSL has been histologically proven to lead to compression of the SSN even without evident neuropathy-associated symptoms. 26

The mean middle-transverse diameter was also calculated after the SNs were distinguished based on their content. The diameter for type-I notches was 8.64 ± 3.36 mm, 8.86 ± 3.12 mm for type II, and 14.05 ± 1.02 mm for type III. Similar were the findings at a study performed by Polguj et al that reported middle-transverse diameter 7.68 ± 2.34 mm for type I, 7.98 ± 2.29 mm for type II, and 9.17 ± 2.63 mm for type III. In that study, middle-transverse diameter was calculated on computer tomography scanning of scapulae. 14 The fact, no statistically significant difference was found between the different types of SN diameters which indicates that the diameter is not bigger in an SN type III in which both suprascapular vessels pass through the notch with the SSN, and as a result, an SSN compression inside the canal may be more likely to occur.

## Limitations of the Study

The present study is anatomically orientated, based on cadaveric material. It was strictly focused on the possible clinical impact of STSL and SN morphological features and SN's contents. For this reason, other morphometrical features of the scapulae dissected were not taken under consideration. Also, demographical characteristics of the donors, such as gender age, weight, and height, are not presented in this study. Finally, another limitation of our study might be the relatively small sample size.

## Conclusion

There are numerous anatomical conditions that may predispose to SSN compression and resulting neuropathy, some of which are SN shape and STSL ossification. The number of elements passing beneath the STSL seems not to play a significant role in the SN's width and area and thus, it could be assumed that SSN compression is more likely to happen when it is accompanied by both suprascapular vessels under the STSL, given that the same area is occupied by three rather than one element or two elements. However, a limitation of our study might be the sample size that has to be evaluated to strengthen our hypothesis to have statistical significance. In any case, when performing an arthroscopic release at the SN, the surgeon has to identify carefully its contents, thus leading to fewer cases of inadvertent structures' damage.
